# Expression of Concern: LINC00152 down-regulated miR-193a-3p to enhance MCL1 expression and promote gastric cancer cells proliferation

**DOI:** 10.1042/BSR-2017-1607-T_EOC

**Published:** 2022-11-18

**Keywords:** gastric cancer, LINC00152, MCL1, miR-193a-3p

The Editorial Office has been made aware of potential issues surrounding the scientific validity of this paper, hence has issued an expression of concern to notify readers whilst the Editorial Office investigates. It has been noted that there are similarities between images in this paper and those found in two papers published by unrelated authors. The mouse tumour images from Figure 3A appear to be very similar to those found in Figure 7A of Zhao et al. 2018 (doi: 10.1111/jcmm.13688) and Figure 7A of Xue et al. 2018 (doi: 10.1111/jcmm.13604). The Figure 5B BGC-823 NC and mimics+linc00152 panels also appear to be duplicated.

